# Anorectal melanoma: surgical management guidelines according to tumour thickness

**DOI:** 10.1038/sj.bjc.6601409

**Published:** 2003-11-25

**Authors:** G H Weyandt, A O Eggert, M Houf, F Raulf, E B Bröcker, J C Becker

**Affiliations:** 1 Department of Dermatology, Julius Maximilians University Würzburg, Josef Schneidr Str. 2, Würzburg 97080, Germany; 2 Department of Coloproktology, St Josefs Hospital, Wiesbaden, Germany; 3 Department of Coloproctology, Raphaelsklinik, Münster, Germany

**Keywords:** abdomino-perineal resection, wide local excision, tumour thickness, local tumour control

## Abstract

Management of patients with anorectal melanoma is still controversial. To reach a rationale therapeutic approach, we reviewed our experience obtained over the past decade. In all, 19 consecutive patients with the diagnosis of anorectal melanoma were included in this retrospective survey. Details of the patients' presentation, symptoms, tumour size and histology and tumour state were recorded, and the primary therapeutic procedures were evaluated in detail. The size of the tumours ranged between 0.5 and 7 cm in diameter. The median tumour thickness was 10 mm (range 0.6–40 mm). At diagnosis, six of 19 patients already presented with either regional or distant metastases. The remaining 13 patients were treated with curative intend, either by abdomino-perineal resection (APR) or wide local excision (WLE). The form of operative therapy, however, had no impact on overall survival. Nevertheless, the incidence of local recurrences was lower after APR even for patients with less favourable tumours. In conclusion, WLE alone is not sufficient for local tumour control of thick anorectal melanoma.

Anorectal melanoma is a rare form of melanoma that constitutes only 0.4–1.6% of all melanoma manifestations (Wanebo *et al*, 1981). It accounts only for approximately 0.5% of all colorectal and anal cancers (Roumen, 1996). Nevertheless, the anorectum is the most common site for development of primary melanoma in the alimentary tract. Owing to its low incidence, treatment of anorectal melanoma to date is not well defined since it was based on retrospective studies reporting on either a limited number of cases or data collected over long time periods, that is, up to 64 years (Brady *et al*, 1995). Thus, optimal surgical treatment for primary tumours is still controversial. This is particularly true if overall survival is not the sole end point, but local tumour control is also taken into account. The chosen form of therapy should allow the best possible rehabilitation, that is, a low risk of local failure and a life free of pain or individual restriction. For anorectal melanoma, this is the decision between life with a colostomy after abdomino-perineal resection (APR) or the risk of local recurrences with severe symptoms after wide local excision (WLE). Wide local excision is defined as a sphincter saving procedure with a defined margin around the tumour in two dimensions; concerning the depth, safety margins are restricted to the subcutaneous compartment. Thus, the excision is performed up to the internal sphincter muscle and a side margin to the tumour up to 2 cm.

In this respect, it is important to consider the value of APR. Although most patients with anorectal melanoma will die from the disease regardless of the chosen therapeutic strategy, APR may provide substantial benefits by avoiding severe symptoms caused by local recurrences, such as incontinence and continuous bleeding of the tumour, which may require further surgical interventions. The goal of our study was to evaluate APR and WLE for management of anorectal melanoma with respect to local tumour control.

## MATERIALS AND METHODS

**Figure 1:**
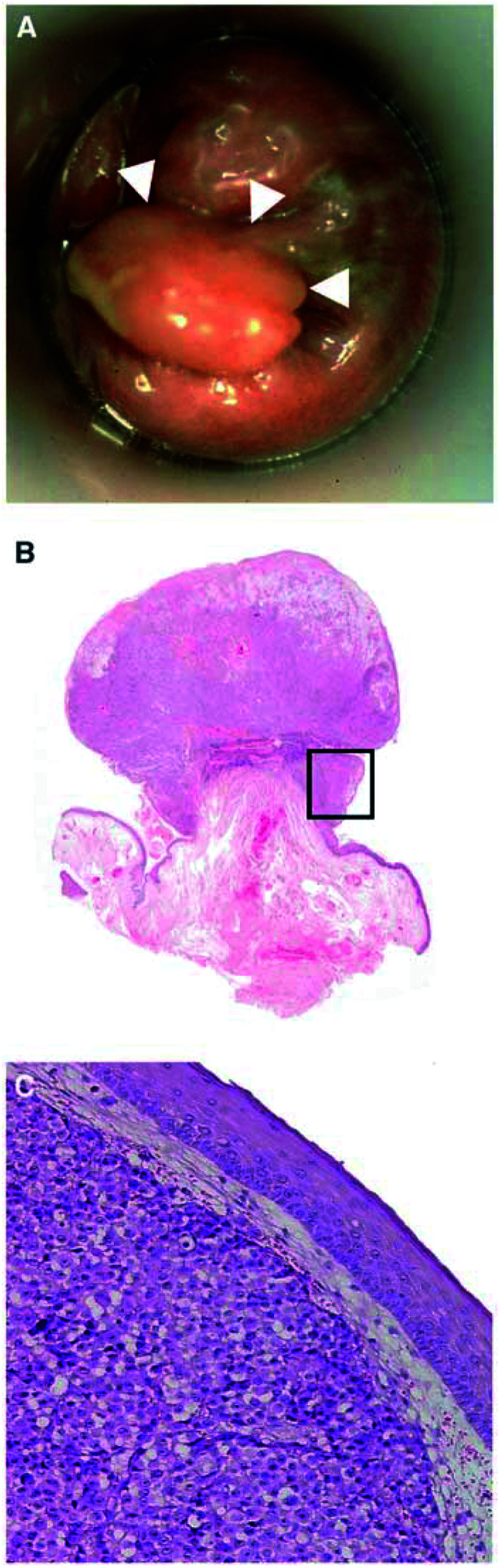
(**A**) A 57-year-old women with a polypoid anal melanoma at 3° with little pigment at the base of the polypoid tumour (arrow). (**B**) Low-power view of polypoid MM of the anorectal region with superficial ulceration (HE × 80). (**C**) *Detail*: Extensive tumour growth beneath intact surface squamous epithelium (HE × 250).

Between 1992 and 2001, 19 patients with the diagnosis of anorectal melanoma were treated at the St Josefs Hospital in Wiesbaden, the Raphaelsklinik in Münster and the University Hospital of Würzburg. Patients were considered to have primary melanoma of the anorectal region when the tumour arose in the rectum, anal canal or anal margin. Figure 1

depicts the typical macroscopic and histologic morphology of anorectal melanoma. In the upper right quadrant of the protological view (arrow), a little pigmented anoderm is obvious, while the complete papillomatous tumour is melanoma.

Patients, who's clinical records showed no clinical evidence of residual tumour postresection, were interpreted as cured. Details of the patients' presentations as well as tumour state, size and depth were recorded by clinical description and the histopathologic reports. Follow-up data were obtained for all 19 patients to the point of death or until February 2003 (15–119 months). The use of statistical methods was renounced due to the limited number of patients in the different sub-groups.

## RESULTS

The patient cohort consisted of nine females and 10 males. Mean age at diagnosis was 62 years (range 33–87 years). Initial symptoms leading to diagnosis included bleeding (*n*=11/58%), sensations of the presence of a mass (*n*=4/21%) or pruritus (*n*=3/16%) (Table 1

**Table 1:** Symptoms at first presentation, *n*=19

**Symptom**	**No. of patients (%)**
Rectal bleeding	11 (58)
Pruritus	4 (21)
Mass	3 (16)
Anal pain	2 (10)
Inguinal mass	2 (10)
Meteorism	2 (10)
Incontinence	1 (5)

**Table 2:** Recurrence distribution after curative treatment

**Localisation**	**No. of patients^a^**
Liver	4
Lung	4
Pelvic	1
Local plus systemic	1
Isolated local	4
Inguinal lymph node	1

a Some patient may have recurrence at more than one site.

). For two patients, the melanoma was discovered upon pathologic review of haemorrhoidectomy.

Of the 19 patients, 14 were dead at the time of evaluation; their death could be attributed to progression of the neoplastic disease. Five patients were still alive. The median tumour size was 3.2 cm in diameter (range 0.5–9 cm) for the total group. The median tumour size in patients with resectable disease was 3.1 cm in diameter (range 0.5–5 cm). Median tumour thickness was 10 mm for the whole cohort (range 0.6–40 mm). Notably, median tumour thickness in patients with resectable disease undergoing APR was 17 mm (range 10–40 mm/*n*=5). This was compared to a tumour thickness of 8.5 mm (range 0.6–10 mm/*n*=8) in patients treated by WLE. Treatment and survival according to tumour depth is summarised in (Table 3

**Table 3:** Treatment and survival according to tumour depth

**Depth (mm)**	**Number**	**Local excision**	**APR**	**Palliative**	**Median survival (months)**
0–1	1	1 (alive)			119
1–4	1	1 (alive)			51
4–10	8	5 (two alive)	1 (one alive)	2	20
10–20	7	1	3	3	23
>20	2		1	1	12

APR=abdomino-perineal resection.

).

At the time of diagnosis, six patients already suffered from clinically evident metastatic disease (Table 4

**Table 4:** Tumour depth according to filiae at diagnosis

**No.**	**Depth (mm)**	**Location**
1	6	Inguinal lymph node
2	10	Inguinal lymph node
3	11	Liver
4	12	Inguinal lymph node
5	40	Lung, inguinal lymph node
6	20	Liver, kidney

). All but one of these patients died within 14 months (mean survival 9 months). The exception was a 56-year-old man, who survived 38 months after local excision with liver and renal metastasis. In three of these stage IV patients, it was necessary to place a palliative stoma to control complications caused by local tumour growth and bleeding.

The 13 patients, treated with curative intent could be divided into two groups: five patients undergoing an APR and eight patients treated by WLE. Of the five patients treated by APR, two died after 17 months, the third after 19 months; the fourth patient had a tumour recurrence in the pelvis after 38 months, which was treated by resection. He died from tumour progression 23 months later. The fifth patient remained tumour free for 24 months after APR.

Of the eight patients treated by WLE, one died after 8 months with systemic tumour recurrence. Five patients (62.5%) presented themselves with local recurrences; in three of them it was detected in an early state without any clinical symptoms, but two suffered from massive tumour growth with persistent bleeding. Three of these patients were treated by APR; in two patients a second WLE was performed. Of the latter, one needed a palliative stoma due to incontinence and he died within 9 months; the other patient was a 59-year-old women, who presented with local tumour recurrence after 36 months, but she did not develop any additional recurrence in the subsequent follow up of 83 months.

Eight of the 13 patients with resectable disease died from disease progression at a mean of 25 months (range 14–61 months) (Table 2). Five patients were still alive and tumour free (mean follow-up of 33 months (range 15–119 months) at the evaluation date.

It was not possible to make a statistical statement about long-term survival due to the limited number of patients.

## DISCUSSION

The prognosis of anorectal melanoma as for most other mucosal presentations of melanoma is poor (Chung *et al*, 1980; Kato *et al*, 1987; Going *et al*, 1989). This poor prognosis seems to be due to the late detection of the disease. Symptomatic tumours are normally large in size and show a deep penetration. Thus, up to one-third of the patients already suffer from dissemination at the time of diagnosis (16–33%) (Thibault *et al*, 1997). This notion reflects our own observations with 32% of the patients suffering from metastatic disease at the time of diagnosis. For these patients there is currently no established therapeutic approach, which allows a favourable outcome.

With regard to patients undergoing surgery with a curative intent, our study further confirms the poor prognosis reported from previous series. Currently, there is no firm recommendation towards either conservative, that is, WLE, or radical, that is APR, surgical approaches. Meta-analyses with the compiled data of 426 patients failed to show the advantage of either approach with respect to overall survival (Thibault *et al*, 1997). It should be noted, however, that in most studies it is difficult to compare patient's prognostic factors due to the fact that insufficient information is provided. Nevertheless, there was a trend that local disease is more effectively controlled by APR than WLE (Abbas *et al*, 1980; Ross *et al*, 1990; Roumen, 1996; Thibault *et al*, 1997). To this end, Thibault reported that isolated local recurrences after APR were rare (Thibault *et al*, 1997). In all, 10 long-term survivors were reported by Brady in a cohort of 85 patients; nine of them received an APR; thus, advocating APR (Brady *et al*, 1995). The mean tumour depth in patients with resectable disease undergoing APR was 7.5 mm (range 0.5–20) compared with 6 mm (range 4–9) in the group of patients treated with local procedures. However, this study is flawed by several inconsistencies such as missing progress reports or the fact that none of the locally treated tumours were below 4 mm in tumour thickness. Roumen (1996) reported a series of 34 patients, 18 of them were treated by APR whereas 16 patients received WLE. Interestingly, only one of the patients treated by APR developed a local recurrence. In contrast, local recurrences were observed in most of the patients (12 of 16) who underwent WLE. Seven of these 12 patients also presented a systemic recurrence. Unfortunately, only for 60% of the patients the histological tumour thickness was given. Ballo *et al* (2002) reported that adjuvant radiation of the pelvis and inguinal lymph nodes after WLE showed similar rates of local tumour control as APR. From 23 patients with mean histological tumour thickness of 5 mm (range 0.3–35 mm), five of them had a local and three a nodal failure, but none of these patients required an APR for palliation.

Anorectal melanoma may represent a systemic disease at the time of diagnosis in most cases, and the choice of the surgical or other local procedures to amend the primary tumour has no influence on the systemic course of the disease.

Thus, therapeutic strategies should be adjusted to the prognosis of the disease. Unfortunately, prognostic parameters for anorectal melanoma remain to be defined. Only a few studies addressed this pertinent question. Goldman *et al* (1990) reported a correlation between overall survival and the size of the tumour. Among 33 evaluated patients only two were long-term survivors. These two patients were characteriszed by a tumour size of less than 2 cm in diameter. The tumour thickness was below 2.5 mm. Unfortunately, in this study the histopathologic data were not reported for all patients.

In contrast, Slingluff *et al* (1990) suggested that the only predictive factor for survival was stage of disease, that is, local, regional or systemic, at the time of diagnosis, whereas Ballo *et al* (2002) reported a favourable disease-free survival outcome when the tumour thickness was below 4 mm.

Hence, anorectal melanoma seems to be similar to cutaneous melanoma for which stage of disease and tumour thickness are the main prognostic factors. For cutaneous melanoma these factors are considered essential to plan further therapeutic procedures. If anorectal melanoma – maybe only anal melanoma – is in this respect indeed comparable to cutaneous melanoma, it should be handled accordingly; thus, the decision between WLE and APR should be governed by the tumour thickness. Preoperative measurement of the tumour thickness may prove to be a valuable tool to plan the operation.

The aim for surgical therapy of anorectal melanoma is to maximise quantity and quality of life. Based on this aim, in early disease with a tumour thickness below 1 mm, a local sphincter saving excision with 1 cm safety margin would be appropriate. In the cases of a tumour thickness between 1 and 4 mm, a local sphincter saving excision with a safety margin of 2 cm seems to be adequate, if the internal sphincter muscle is not afflicted. With respect to local tumour control, WLE alone does not seem to be adequate treatment for anorectal melanomas with a tumour thickness above 4 mm. These patients would benefit from an APR or additional measures to control local tumour reoccurrences in combination with a WLE. If such patients decide to have only a WLE, they should be aware that local failure is common and complications requiring additional surgery may occur. In this respect, adjuvant radiation has been suggested to hold the promise of sphincter preservation together with sufficient tumour control (Ballo *et al*, 2002). However, acute radiation-related dermatitis was noted by most patients and chronic radiation-related toxicity occurred in six of 23 patients.
